# Development of an educational mobile application for patients submitted to orthognathic surgery

**DOI:** 10.1590/1518-8345.2904.3143

**Published:** 2019-07-18

**Authors:** Cristina Silva Sousa, Ruth Natalia Teresa Turrini

**Affiliations:** 1 Universidade de São Paulo, Escola de Enfermagem, São Paulo, SP, Brasil.

**Keywords:** Mobile Application, Smartphone, Telemedicine, Patient Education as Topic, Orthognathic Surgery, Perioperative Nursing, Aplicativos Móveis, Smartphone, Telemedicina, Educação de Pacientes como Assunto, Cirurgia Ortognática, Enfermagem Perioperatória, Aplicaciones Móviles, Teléfono Inteligente, Telemedicina, Educación del Paciente como Asunto, Cirugía Ortognática, Enfermería Perioperatoria

## Abstract

**Objective:**

to develop, evaluate and correlate the acceptability of an educational mobile application to patients submitted to orthognathic surgery.

**Method:**

methodological study based on systematic instructional design with contents aimed at patient learning through a mobile application. Usability and user satisfaction were evaluated by 30 patients in the perioperative stage through an electronic questionnaire sent by social networks, e-mail and business card, measured using the System Usability Scale instrument validated in Portuguese and user satisfaction with an instrument based on another study, after its applications. Data were analyzed with descriptive statistics and Spearman correlation.

**Results:**

the application named “OrtogApp” features content validated in a previous study included five learning content sessions essential for managing perioperative care, and it is available on IOS and Android platforms. Usability corresponded to 79.8 + 15.4 points and the satisfaction index was 82.9%; correlation of age, schooling and uses of the application with the instruments was not significant.

**Conclusion:**

OrtogApp is an educational application with content validated by professionals, resulting in high user satisfaction and good usability. Patients may use the application as supportive educational material to supplement guidance provided by perioperative nurses and/or surgeons during perioperative care.

## Abstract (pt)

**Objetivo:**

desenvolver, avaliar e correlacionar a aceitabilidade de um aplicativo educativo de celular para pacientes submetidos à cirurgia ortognática.

**Método:**

estudo metodológico baseado no *design* instrucional sistemático com conteúdo de aprendizagem ao paciente por aplicativo de celular. Usabilidade e satisfação do usuário foram avaliadas por 30 pacientes em perioperatório por meio de questionário eletrônico enviado por redes sociais, *e-mail* e cartão de visita, mensurados com o instrumento *System Usability Scale* validado em português, e satisfação do usuário com instrumento baseado em outro estudo, utilizado após uso do aplicativo. Os dados foram analisados com estatística descritiva e correlação de Spearman.

**Resultados:**

o aplicativo nomeado “OrtogApp” apresenta conteúdo validado em estudo prévio, incluiu cinco sessões de conteúdo de aprendizagem essenciais para gerenciar o cuidado perioperatório e foi disponibilizado nas plataformas IOS e Android. Usabilidade correspondeu a 79,8 + 15,4 pontos e o índice de satisfação 82,9%. A correlação da idade, escolaridade e usos do aplicativo com os instrumentos não foi significativa.

**Conclusão:**

OrtogApp é um aplicativo educativo com conteúdo validado por profissionais que resultou em alta satisfação do usuário e boa usabilidade. Os pacientes podem utilizar o aplicativo como material educativo de apoio complementar às orientações dadas por enfermeiras perioperatórias e/ou cirurgiões durante o cuidado perioperatório.

## Abstract (es)

**Objetivo:**

desarrollar, evaluar y correlacionar la aceptabilidad de una aplicación educativa de celular para pacientes sometidos a la cirugía ortognática.

**Método:**

estudio metodológico basado en el diseño instruccional sistemático con contenidos de aprendizaje al paciente por una aplicación de celular. Facilidad de uso y la satisfacción del usuario se evaluaron para 30 pacientes en el perioperatorio mediante un cuestionario electrónico enviado por redes, correo electrónico y tarjeta de visita social. La medida del instrumento Escala usabilidad Sistema validado en el instrumento portugués y la satisfacción del usuario basado en otro estudio fueron aplicados después del uso de la aplicación. Los datos fueron analizados con estadística descriptiva y correlación de Spearman.

**Resultados:**

la aplicación denominada “OrtogApp” presenta contenido validado en estudio previo, incluye cinco sesiones de contenido de aprendizaje esenciales para administrar el cuidado perioperatorio. Era disponible en las plataformas IOS y Android. La facilidad de uso correspondió a 79,8 + 15,4 puntos y el índice de satisfacción fue 82,9%, la correlación de la edad, escolaridad y usos de la aplicación con los instrumentos no fue significativa.

**Conclusión:**

OrtogApp es una aplicación educativa con contenido validado por profesionales, que resultó en alta satisfacción del usuario y buena facilidad de uso. Los pacientes pueden utilizar la aplicación como material educativo de apoyo complementario a las orientaciones dadas por enfermeras perioperatorias y/o cirujanos durante el cuidado perioperatorio.

## Introduction

There is a current growing movement in mobile technologies and applications that collaborate to build a new modality of health care. A systematic review aimed at identifying the use of smartphone applications in the health area retrieved 39 studies that were categorized into eight domains: diagnosis (n = 11), telemedicine (n = 9), surgical simulator (n = 6), training (n = 5), data collection (n = 3), patient education (n = 2), behavior (n = 2), and surgical planning (n = 1)^(^ ^1^ ^)^.

There are no review studies targeting the use of educational applications for surgical patients. Two studies^(^ ^2^ ^-^ ^3^ ^)^ described applications as a resource for preoperative preparation and obtained satisfactory results. However, no publication portrays patients of the maxillofacial specialty.

Using technology in the education of surgical patients represents an evolution in nursing care. Applications are a resource capable of expanding access to information, since smartphones and access to the internet have become popular.

A study aimed at evaluating the use of an application to increase the knowledge of surgical patients about safety in general, urology, orthopedics and neurology surgeries observed a significant increase in patient knowledge resulting from the use of this tool^(^ ^4^ ^)^.

Despite studies on surgical preparation, guidance or monitoring, there are no studies on applications as a complementary resource for health education. The present study describes an educational application that is ideal to facilitate access to information and to increase the number of patients with access to content to assist in the management of self-care during the perioperative period. It is the first mobile application developed by nursing and focusing on surgical patients.

The postoperative period of orthognathic patients lasts around two months and requires management of self-care with respect to oral hygiene, feeding, pain, opening of the oral cavity, resting, and control of facial edema. Patients need clear guidance on how to perform post-operative care, and the use of educational materials as an auxiliary method to reinforce verbal guidance has shown effective results^(^ ^5^ ^-^ ^7^ ^)^.

Due to the absence of educational applications for surgical patients with the aim of complementing verbal and auxiliary guidance about self-care at the home context, and considering the postoperative complexity of orthognathic surgery, this study aimed to develop, evaluate and correlate the acceptability of an educational application (app) for patients submitted to orthognathic surgery.

## Method

This is a methodological study based on the systematic instructional design (SID). This study was approved by the Ethics and Research Committee of the School of Nursing of the University of São Paulo CAAE under Opinion: 67081317.2.0000.5392.

The systematic instructional design was developed in 1978 and is one of the most widespread methods in the world. It covers the stages of analysis, design/development, implementation and evaluation^(^ ^8^ ^-^ ^9^ ^)^.

In the first stages of analysis and design, the content and scope of the project were based on the educational material “Orthognathic Surgery for Patients”, which was constructed by the researcher and validated in a previous study^(^ ^10^ ^)^. The existing smartphone applications for patient education were also analyzed.

The design was based on the following learning contents: acquisition of knowledge about the perioperative stage of orthognathic surgery by means of smartphones as motivational incentive for self-care implementation and support in postoperative recovery at home.

**Figure 1:**
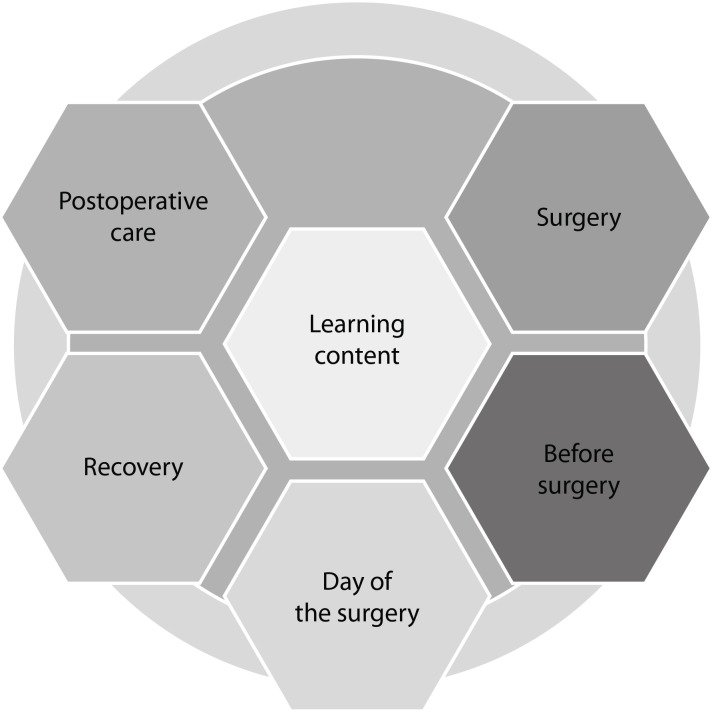
Learning contents about the perioperative phase of orthognathic surgery. São Paulo, SP, Brazil, 2017

The contents for learning about perioperative orthognathic surgery are presented in five sessions in Figure 1.

The objectives included in each learning session include in “surgery”: knowing the procedure, indications, and surgical techniques; before surgery: familiarization with the need for the procedure; day of the surgery: review of the preoperative preparation and resources needed for hospitalization; recovery: understanding the recovery phase of the surgery and possible complications; postoperative care: knowing the postoperative care at home to manage self-care and help in the recovery of the surgery.

In the development phase, based on the objectives, in the learning contents and in the idealized structure, the educational application called “OrtogApp” was idealized. Learning content was organized considering the objectives and scope of learning based on a learning menu flowchart, and the version of an educational application was developed by a web professional in a prototype visible in the Ionic View^®^ app, so that the researcher could visualize the design before its official publication.

The content was arranged to be viewed comfortably. After startup, the icons with images appear on the screen, and by tapping the icon, the user has access to the sub-contents. A “back” icon is inserted in the contents to return the main screen. Parts of text that deserve greater attention from the reader are highlighted and have a different color. Images were inserted into the content to draw the reader's attention and elucidate the text.

**Figure 2:**
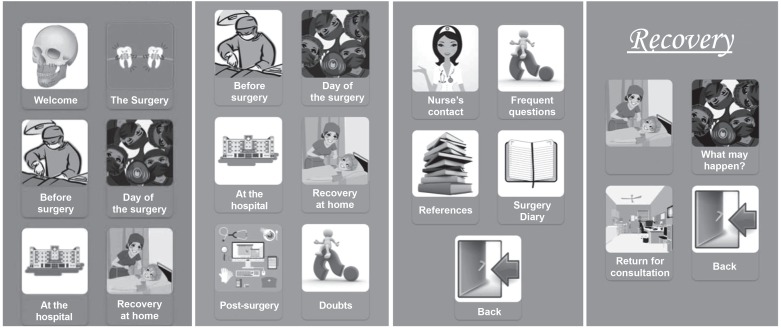
Access menu and sub-contents navigated by icons. São Paulo, SP, Brazil, 2017

The learning contents and sub-contents of the educational material are: surgery (what is surgery, indication, different surgical techniques); before surgery (type of procedure, need for preoperative exams); day of the surgery (guidance on fasting, items to take to the hospital, clothing, documents); recovery (how the recovery takes place, possible complications, return to the medical office); postoperative care (guidance on oral hygiene, oral diet, mouth opening, ice application, facial exercises, lip moisturizing, sun exposure, bathing/dressing, pain, rest, sleeping/breathing); other items: presentation (application presentation), hospital (hospital routine: admission, anesthesia recovery, hospital discharge), and doubts (frequently asked questions of social networks, link for sending e-mail to specialist nurse, logbook) (Figure 2).

Icons are displayed in the images as well as in written text. Once the icon is activated, the reader is directed to a page with its sub-contents with information about the perioperative period of orthognathic surgery and self-care post-discharge.

In the implementation and dissemination phase, the “OrtogApp” application was designed for IOS and Android systems. The operational platforms (IOS/Android) perform an evaluation of the app before it is published and made available for download.

In this process, the App Store returned with a message saying the application was simple and required more interactivity to be more attractive, which led to the reconstruction of the design and functions of the app. The functions of contact with specialist nurse to clarify doubts and geographical locator of possible points to access medical service were inserted.

The item contact with specialist nurse triggers the email manager of the user's smartphone and the doubts sent are answered within 48 hours by email. The geographic locator triggers google maps. After approval of the App Store, the app was made available for free download on both platforms (IOS/Android), and the patent was registered as a computer program at the National Institute of Industrial Property (INPI).

The app was disseminated in some maxillofacial surgery clinics of the city of São Paulo, and the application was presented on social networks to increase the use and the possibility of national evaluation.

For dissemination in medical offices, a business card was made containing the presentation of the App, an invitation to participate in the research with QR code, and a link for accessing the research instrument; the card was offered to patients by the surgeons in their offices. This card was also offered in hospital units by the researcher.

In social networks, the app and the invitation to participate in the research with the link to access the data collection instrument were presented through a post in virtual communities. Direct messages were also sent to patients who searched for the theme “orthognathic surgery” with presentation of the app, invitation to participate, and link of the research instrument. Invitations were repeated every fortnight during the data collection period.

**Figure 3:**
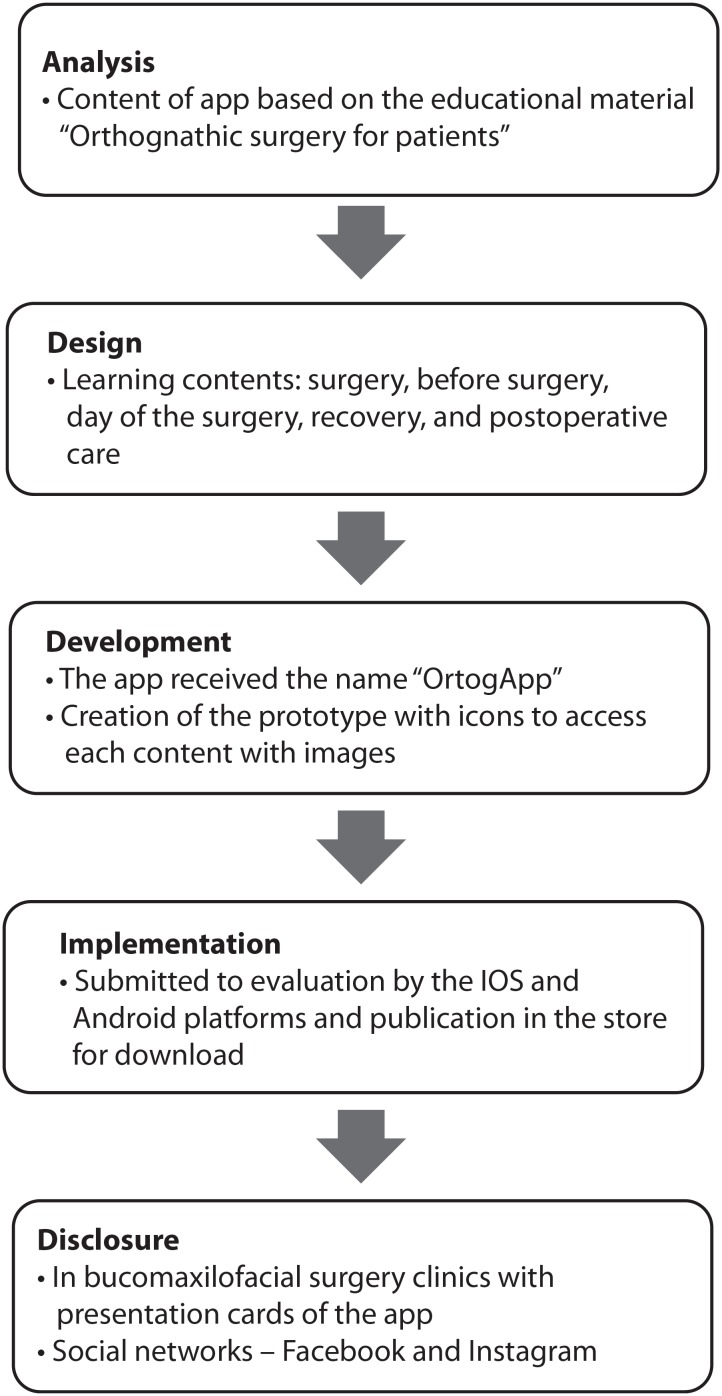
Steps taken to develop the app. São Paulo, SP, Brazil, 2017

The informed consent term was inserted in the research instrument; the non-acceptance of the respondent automatically terminated his/her participation. A review of the route taken to develop the app can be seen in Figure 3.

After starting the use of the app, its usability and the satisfaction of users of the OrtogApp were evaluated.

Usability is defined as the potential to be used in software engineering - product quality. Usability is a set of attributes of the software that are based on the effort required for using it and individual evaluation of such use for an implicit set of users^(^ ^11^ ^)^.

The instrument used to evaluate usability was the System Usability Scale (SUS) in a translated and validated version in the Portuguese language^(^ ^12^ ^)^. The instrument had ten questions and a five-point Likert scale with values varying from 1 (strongly disagree) to 5 (fully agree), where 3 means neutral. There were five positive statements (items with odd numbers) and five negative statements (items with even numbers) that are changed. The overall usability value of the system can range from 0 to 100 points; 0 indicates extremely poor usability and 100, excellent usability. Values over 68 points reflect acceptable usability^(^ ^13^ ^)^.

The evaluation items include: I would like to use the product frequently; complexity of the product; ease of use; help is necessary to use it; the features were well integrated; there were many inconsistencies; it is possible to learn quickly how to use the product; it is complicated to use; confidence in using the product; learning is necessary before dealing with the product.

The instrument to evaluate satisfaction was based on an instrument of another study^(^ ^14^ ^)^ for evaluation of applications, which consists of eight items and a five point Likert scale with values varying from 1 (very dissatisfied) to 5 (very satisfied), where 3 means neutral. This instrument is based on the Experience Sampling Method (ESM) technique, which makes it possible to measure two dimensions: the type of emotion (positive or negative) and the intensity of emotion; the sum of its values are converted into percentage of satisfaction.

The items for evaluation were: downloading and using the application; with the use of the application; handling the application; use of the app in the daily routine; functionalities; feeling about using again the application; communicability; help made available - contact a nurse and logbook.

To evaluate the acceptability of the app, a total of 30 patients in the pre or postoperative period of orthognathic surgery were contacted and consulted about the interest in accessing and evaluating the application through an electronic questionnaire, in order to obtain a feedback about its usability and satisfaction of the user with the new tool to support the perioperative care of orthognathic surgery.

The population sample was defined by convenience, considering the recommendation of the usability tests. For usability tests, a sample of 8 to 25 participants is a reasonable interval^(^ ^15^ ^)^.

Data were inserted in an Excel® worksheet and analyzed for demographic characterization according to descriptive statistics with absolute values, means, and standard deviations. Values of the SUS scale are calculated according to the item; for odd items, the value is obtained by subtracting 1 from the scale position, and for even items, by subtracting 5, summing all items and multiplying by 2.5 to obtain the global value of usability of the system that can range from 0 to 100 points.

For user satisfaction assessment, the scores obtained from the respondents per each question were summed and transformed into percentages. The variables age, schooling and use of the application were submitted to the Spearman correlation test with SUS and satisfaction scores. Type I error was set at 5% as statistically significant (p < 0.05).

## Results

After making the app available for download, the Google Play platform accounted for 66.7% of downloads, followed by 33.3% of the App Store. Based on data from November/2017 to May/2018 by the development platform manager, there were 447 installations through Google Play and 206 through the App Store.

The patients who evaluated the application had a mean age of 29.7 ± 7.3 years; the majority had complete higher education (n = 10, 33.3%), were from the southeastern region (n = 21.70%), and lived in the state of São Paulo. Regarding the perioperative period, 46.6% (n = 14) were in the preoperative phase and 53.3% (n = 16) were in the postoperative face when they accessed the application. Most participants received information about the app through social networks (n = 16) (Table 1).

**Table 1:** Sociodemographic characteristics and access to the application by the participants. São Paulo, SP, Brazil, 2017

Variables		N	%
Age in years			
	19-30	13	43.3
	31-39	15	50.0
	>40	2	6.7
Schooling			
	Complete High School	6	20.0
	Complete Higher Education	10	33.3
	Incomplete Higher Education	9	30.0
	Post-graduation *lato sensu*	5	16.7
Brazilian regions - Download			
	North	-	-
	Northeast	3	10.0
	Midwest	2	6.7
	Southeast	21	70.0
	South	3	10.0
Perioperative period			
	Preoperative –planning of the surgery	7	23.3
	Preoperative - near the date of surgery	7	23.3
	Postoperative - up to one week	4	13.3
	Postoperative - more than one week	10	33.3
	Postoperative - more than six months	1	3.3
	Postoperative - more than twelve months	1	3.3
How obtained information about the app			
	App Store	1	3.3
	Social networks	16	53.3
	Heard about the app	2	6.7
	Surgeon's indication	2	6.7
	Friend's indication	9	30.0

The SUS instrument averaged 79.8 + 15.4, thus indicating good with respect to usability. Of these 73.3% (n = 22) scored higher than 68, value considered the cutoff score of the instrument, and 26.6% (n = 8) scored between 50 and 67 points, bordering but still acceptable. Scores below 50 are considered to indicate no usability. The correlation of usability scores and the variables age, schooling, and use of the application, tested by the Spearman correlation test, were not significant. Age (p = 0.804), schooling (p = 0.793), and use of the application (p = 0.673).

**Figure 4:**
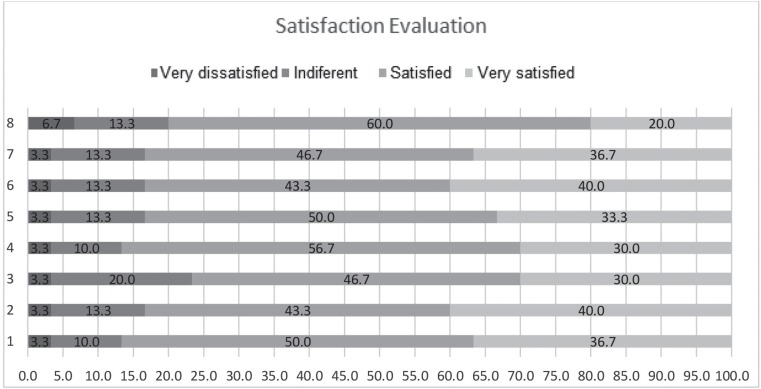
Evaluation of user satisfaction presented by items of the instrument. São Paulo, SP, Brazil, 2017

The frequency of accesses to the application was 40% (n = 12) two to three times, followed by 30% (n = 30) once, 20% (n = 6) more than five times, and 10% (n = 3) five times. The user satisfaction corresponded to the average of 24.9 + 1.0 users corresponding to 82.9%. Satisfaction indexes per question are presented in Figure 4; there was no one case of evaluation as “unsatisfied” in the Likert-type scale.

The correlation of the variables age, schooling, and use of the application with the level of satisfaction tested by the Spearman correlation test were not significant. Age (p = 0.798), schooling (p = 0.281), and use of the application (p = 0.428).

## Discussion

This is the first educational smartphone application for patients in Portuguese, built by a perioperative nurse. The use of this technology allows patients to access an educational material that complements the guidelines provided by professionals in the perioperative period and offers the possibility of contact with a specialist nurse during their surgical procedure.

Mobile devices are present in many aspects of our lives and offer fast, adaptive solutions to day-to-day tasks. In the health area, applications have gained prominence, either those developed for professionals or for patients, and can be used to inform, instruct, record, display, guide, remind, or alert and communicate^(^ ^16^ ^)^.

OrtogApp offers information and interaction through electronic communication (e-mail) between professionals and patients. Regarding usability and level of user satisfaction, the findings of this study reflected good usability and high user satisfaction with the educational application.

Usability results were lower than a study conducted in the US where guidance was given to 15 patients undergoing colorectal surgery, which reached a score of 95^(^ ^17^ ^)^. The present results were similar to another study with 45 patients undergoing colorectal surgery with a score of 87 points^(^ ^18^ ^)^. Indexes above 70 points are considered good values for usability and acceptability of the user, and over 85 are considered excellent^(^ ^13^ ^)^.

In this study, higher usability and satisfaction scores were observed among individuals between 31 and 39 years old, individuals with superior education, and among those who had higher number of repetitions in the use of the application. A systematic review on usability and efficacy of applications for diabetes patients identified the main usability problems: multi-step tasks, limited functionality and interaction, and difficult system navigation^(^ ^19^ ^)^.

OrtogApp is easy to navigate, it does not require task execution by the patient, but has limited interaction (e-mail only), a format that makes instant response impossible, different from instant messaging applications.

The use of this application with accessible language allows the patient to understand and to be empowered as to preparation and management of self-care during the postoperative period. Having access to the application at any time can prevent the patients from searching for wrong information on social networks and on the internet, compromising their recovery.

In the study where the efficacy of the educational material, the content of OrtogApp, was evaluated, the findings of the randomized clinical trial showed an increase in the knowledge of patients in the postoperative period with the use of the material, although anxiety levels were not reduced^(^ ^20^ ^)^.

In an American educational application with 20 patients undergoing bariatric surgery, user satisfaction was high with respect to this application^(^ ^21^ ^)^. It was observed an increase in the knowledge and commitment of the patients and the tool was useful for the preparation of surgical patients.

Another application with 13 mastectomized women obtained only 46.2% for good and 38.5% for excellent^(^ ^22^ ^)^. The use of the application reduced the anxiety and depression levels measured by the Hospital Anxiety and Depression Scale (HADS) on the 7^th^ postoperative day.

In a clinical trial, the use of an application with guidance on the preparation for colonoscopy showed that the patients using the application had better intestinal preparation than patients who received the verbal guidelines^(^ ^23^ ^)^.

Mobile applications have proved to be beneficial for patient care; nurses can use this feature as an ally of their perioperative guidance, recommending the patients to use the application as a method of consultation and interaction with professionals.

The largest number of downloads in the Google Play store reflects the characteristic of Brazil, with more users using the Android system instead of IOS. This difference is due to the higher cost of smartphones with IOS system compared to Android.

Regarding user access to the educational application, most of the respondents reported to have received information through social networks, followed by friend's indication. In an epidemiological study on the use of health-related mobile applications, it was shown that the applications were more often shared in social networks (29%) and less frequently among health service providers (17%)^(^ ^24^ ^)^, showing similarity to the findings of the present study.

Among the implications for clinical practice, applications need to be refined to meet patient demand, and nurses should include them into their daily practice, recommending and managing the use of this feature as part of their routine health management. Nurses should ensure that the applications they recommend to patients have validated evidence-based information.

For future studies, it is recommended to develop more applications aimed at other surgical procedures, expanding the specialties. It is also necessary to validate instruments that can evaluate the quality of the available applications, allowing the qualification of the application and recommending to the patient the one that best meets their needs.

Among the limitations of the study are the availability of the app in only one language and its restriction to the surgical procedure. Despite widespread use in social networks and clinics, a wider dissemination of the app, covering all oral and maxillofacial surgeons in the country is necessary for the national reach of the product, since the greatest demand of use came from the city of São Paulo.

## Conclusion

The application developed for patients submitted to orthognathic surgery is an innovative resource for perioperative nursing; it allows the patients to have immediate access to information with content validated by a multiprofessional team, and can work as an application with educational material to complement the guidance provided by nurses in the perioperative stage. The findings of the study resulted in high user satisfaction and good usability, confirming the acceptability of the product by the users.

## References

[1] Mobasheri MH, Johnston M, Syed UM, King D, Darzi A (2015). The uses of smartphones and tablet devices ins urgery: a
systematic review of the literature. Surgery. [Internet].

[2] Mundi MS, Lorentz PA, Grothe K, Kellog TA, Collazo-Clavell ML (2015). Feasibility of Smartphone-based education modules and ecological
momentary assessment\intervention in pre-bariatric surgery
patients. Obes Surg. [Internet].

[3] Kim K, Pham D, Schwarzkopf R (2016). Mobile application use in monitoring patient adherence to
perioperative total knee arthroplasty protocols. Surg Technol Int. [Internet].

[4] Cho S, Lee E (2017). Effects of the smartphone application “Safe Patients” on
knowledge of patient safety issues among surgical patients. Comput Inform Nurs. [Internet].

[5] Fredericks S, Guruge S, Sidani S, Wan T (2010). Postoperative patient education: a systematic
review. Clin Nurs Res. [Internet].

[6] Cinar FI, Tosun N, Akbayrak N, Simsek I, Cinar M, Erdem H (2013). Comparison of the efficacy of written information vs. verbal plus
written information in rheumatic patients who receive colchicine
treatment. Gulhane Med J. [Internet].

[7] Achaval S, Fraenkel L, Volk RJ, Cox V, Suarez-Almazor ME (2012). Impact of educational and patient decision aids on decisional
conflict associated with total knee arthroplasty. Arthritis Care Res. (Hoboken). [Internet].

[8] Patel SR, Margolies PJ, Covell NH, Lipscomb C, Dixon LB (2018). Using instructional design, analyze, design, develop, implement,
and evaluate, to develop e-learning modules to disseminate supported
employment for community behavioral health treatment programs in New York
State. Front Public Health. [Internet].

[9] Barra DCC, Paim SMS, Dal Sasso GTM, Colla GW (2017). Methods for developing mobile apps in health: na integrative
review of the lietarture. Texto Contexto Enferm. [Internet].

[10] Sousa CS, Turrini RNT (2012). Creating and validating educational material for patients
undergoing orthognathic surgery. Asian Nurs Res. (Korean Soc Nurs Sci). [Internet].

[11] Bevan N, Carter J, Harker S, Kurosu M. (2015). ISO 9241-11 revised: What have we learnt about usability since
1998?.

[12] Martins AI, Rosa AF, Queirós A, Silva A, Rocha NP (2015). European portuguese validation of the SUS. Procedia Computer Sci. [Internet].

[13] Bangor A, Kortum P, Miller J (2009). Determining what individual SUS scores mean: Adding an adjective
rating scale. J Usabil Stud. [Internet].

[14] Knonbauer AH, Santos CAS, Vieira V (2012). An experimental study evaluating the experience of mobile application
users from automatic capture of contextual data and interaction.

[15] Macefield R (2009). How to specify the participant group size for usability studies:
a practitioner's guide. J Usability Studies. [Internet].

[16] Ferguson C, Jackson D (2017). Selecting, appraising, recommending and using mobile applications
(apps) in nursing. J Clin Nurs. [Internet].

[17] Scott AR, Aloe EA, Naik AD, Berger DH, Suliburk JW (2017). Mixed-methods analysis of factors impacting use of a
postoperative mHealth app. JMIR Mhealth uHealth. [Internet].

[18] Perorellin N, Fiore JF, Kaneva P, Somasundram A, Charlebois P, Liberman AS (2018). An app for patient education and self-audit within an enhanced
recovery program for bowel surgery: a pilot study assessing validity and
usuability. Surg Endosc. [Internet].

[19] Fu H, McMahon SK, Gross CR, Adam TJ, Wyman JF (2017). Usability and clinical efficacy of diabetes mobile applications
for adutls with type 2 diabetes: a systematic review. Diabetes Res Clin Practice. [Internet].

[20] Sousa CS, Poveda VB, Turrini RNT (2018). Perioperative booklet for orthognathic patients: a randomized
controlled clinical trial. J Nurs Educ Practice. [Internet].

[21] Mundi MS, Lorentz PA, Grothe K, Kellogg TA, Collago-Clarell ML (2015). Feasibility of smartphone-based education modules and ecological
momentary assessment/intervention in pre-bariatric surgery
patients. Obes Surg. [Internet].

[22] Foley NM, O’Connell EP, Lehane EA, Livingstone V, Maher B, Kaimkhane S (2016). PATI: Patient accessed tailored information: A pilot study to
evaluate the effect on preoperative breast cancer patients of information
delivered via a mobile application. Breast. [Internet].

[23] Kang X, Zhao L, Leung F, Luo H, Wang L, Wu J (2016). Delivery of instructions via mobile social media app increases
quality of bowel preparation. Clin Gastroenterol Hepatol. [Internet].

[24] VonHoltz LA, Hypolite KA, Carr BG, Shofer FS, Winston FK, Hanson CW (2015). Use of mobile apps: a patient-centered approach. Acad Emerg Med.[Internet].

